# Differential DNA methylation and metabolite profiling of Atlantic killifish (*Fundulus heteroclitus*) from the New Bedford Harbor Superfund site

**DOI:** 10.1007/s10646-023-02724-w

**Published:** 2024-01-06

**Authors:** Jiwan Kim, Dawoon Jung, Nivedita Chatterjee, Bryan Clark, Diane Nacci, Suhkmann Kim, Jinhee Choi

**Affiliations:** 1 School of Environmental Engineering, University of Seoul, 163 Seoulsiripdae-ro, Dongdaemun-gu, Seoul, 02504 Korea; 2 Korea Environment Institute, Division of Environmental Health, Sejong, 30147 Korea; 3 U.S. Environmental Protection Agency, Office of Research and Development, Center for Environmental Measurement and Modeling, Atlantic Coastal Environmental Sciences Division, Narragansett, RI USA; 4 Department of Chemistry, Center for Proteome Biophysics and Chemistry Institute for Functional Materials, Pusan National University, Busan, 46241 Korea; 5 Present Address: NanoSafety Group, International Iberian Nanotechnology Laboratory, Av. Mestre Jose Veiga s/n, 4715-330 Braga, Portugal

**Keywords:** Atlantic killifish, New Bedford Harbor Superfund site, DNA methylation, Metabolomics, Tissue-specific

## Abstract

Atlantic killifish (*Fundulus heteroclitus*) is a valuable model in evolutionary toxicology to study how the interactions between genetic and environmental factors serve the adaptive ability of organisms to resist chemical pollution. Killifish populations inhabiting environmental toxicant-contaminated New Bedford Harbor (NBH) show phenotypes tolerant to polychlorinated biphenyls (PCBs) and differences at the transcriptional and genomic levels. However, limited research has explored epigenetic alterations and metabolic effects in NBH killifish. To identify the involvement of epigenetic and metabolic regulation in the adaptive response of killifish, we investigated tissue- and sex-specific differences in global DNA methylation and metabolomic profiles of NBH killifish populations, compared to sensitive populations from a non-polluted site, Scorton Creek (SC). The results revealed that liver-specific global DNA hypomethylation and differential metabolites were evident in fish from NBH compared with those from SC. The sex-specific differences were not greater than the tissue-specific differences. We demonstrated liver-specific enriched metabolic pathways (e.g., amino acid metabolic pathways converged into the urea cycle and glutathione metabolism), suggesting possible crosstalk between differential metabolites and DNA hypomethylation in the livers of NBH killifish. Additional investigation of methylated gene regions is necessary to understand the functional role of DNA hypomethylation in the regulation of enzyme-encoding genes associated with metabolic processes and physiological changes in NBH populations.

## Introduction

The non-migratory Atlantic killifish (*Fundulus heteroclitus*) along the American Atlantic coast is able to survive in urban estuaries heavily polluted with a mixture of industrial toxic compounds (Nacci et al. 1999, 2010; Whitehead et al. 2017). Different populations of killifish inhabiting near EPA-designated Superfund sites have rapidly evolved adaptation to local contamination, showing high tolerance to polychlorinated biphenyls (PCBs) and polycyclic aromatic hydrocarbons (PAHs) (Nacci et al. 2010; Whitehead et al. 2017). One of the contaminated urban estuaries on the northeast coast is the New Bedford Harbor (NBH) in Massachusetts. Although industrial waste was directly discharged into the harbor from the 1940s to the 1970s, and PCB discharge was discontinued in 1976 (Bergen et al. 2005; EPA 2011), bioaccumulation of PCBs has been observed in killifish sampled from NBH over 30 years later (Fritsch et al. 2015; Gräns et al. 2015). Analysis of hepatic PCB concentrations showed that the levels of non-dioxin-like and dioxin-like PCBs in NBH fish were 2005–3554 and 272–487 times higher, respectively, compared to those in fish from a reference site (Gräns et al. 2015). In another study, the total PCB concentration in NBH fish was 118,746 ng/g, which was 682 times greater than that in fish from Scorton Creek (SC), a nearby nonpolluted river (Fritsch et al. 2015).

The progenies of wild-caught killifish from PCB- and PAH-contaminated sites showed heritable resistance to dioxin-like compounds (DLCs) exposure compared to fish populations from non-polluted sites across generations (Meyer and Di Giulio 2003; Nacci et al. 1999, 2002). In embryos from tolerant populations, many research groups discovered a dramatic reduction in DLC- and PAH-induced developmental defects (e.g., cardiac deformity, organ malformation, and vascular hemorrhaging), which were clearly observed in sensitive populations from clean sites. Researchers found that the low inducibility of cytochrome P450 1 A (CYP1A) was the hallmark of evolved killifish tolerance (Bello et al. 2001; Whitehead et al. 2017). Furthermore, genomic studies have revealed that genes involved in the aryl hydrocarbon receptor (AHR) signaling pathway play a key role in this adaptive response (Hahn et al. 2004; Nacci et al. 2016; Osterberg et al. 2018; Timme-Laragy et al. 2008; Whitehead et al. 2017). Some studies identified polymorphic variants of both *AHR1a* and *AHR2a* loci and strong signatures of selection between tolerant-sensitive pairs of populations in genomic regions of *AHR1a/2a*, *AIP*, and *CYP1A* in NBH populations (Hahn et al. 2004; Reid et al. 2016). However, other classes of chemicals that do not exert toxicity via the AHR pathway were detected in the polluted sites, and many other genes harboring signatures of selection were also unique to the tolerant killifish (Reid et al. 2016; Whitehead et al. 2017). These results suggest that AHR pathway genes contribute a small proportion of the suite of genes associated with tolerance and that pathways other than the AHR pathway can be involved in the adaptation of killifish.

Epigenetic mechanisms play crucial roles in gene regulation, phenotypic plasticity, physiology, development, and the maintenance of genome integrity, mainly through DNA methylation, chromatin modification, and small non-coding RNA molecules without changing the DNA sequence (Ashe et al. 2021; Cavalieri and Spinelli 2017). Despite the fact that epigenetic states are typically erased and reset at each generation, there is an increasing recognition that epigenetic variations have direct and indirect impacts on evolutionary processes (Ashe et al. 2021). Additionally, DNA methylation, one of the principal epigenetic modulators, can interact with metabolites and control metabolic pathways (Petersen et al. 2014; Thompson et al. 2010; Torres et al. 2019). Therefore, epigenetic mechanisms can contribute to population adaptation, in combination with metabolic alterations. However, there have been few studies regarding epigenetic alterations in response to long-term pollution (Aluru et al. 2011; Glazer et al. 2018; Timme-Laragy et al. 2005), metabolite levels, and metabolic effects in killifish inhabiting polluted environments (Glazer et al. 2018).

This study aimed to identify the involvement of epigenetic and metabolic regulation in the tolerance of evolved killifish to highly contaminated environments. Metabolomics provides a direct signature of biochemical activity and physiological state, which reflects the effects of multiple upstream factors such as the transcriptome and proteome (Gieger et al. 2008; Glazer et al. 2018; Wilmes et al. 2013). We examined differences in global DNA methylation and metabolomic profiles between the NBH and SC killifish populations in brain and liver tissues. In addition to tissue-specific effects, we also explored potential sex-related variations.

## Materials and method

### Fish sample collection

Adult killifish were collected in the fall of 2018 using galvanized steel minnow traps baited with squid at the New Bedford Harbor (NBH) estuarine sites along the Atlantic Coast of the USA, as described in detail elsewhere (Nacci et al. 2010, 2002). The fish were also collected from a nearby reference site, Scorton Creek (SC), in Sandwich, Massachusetts. The fish were sacrificed within 48 h of capture and stored at −80 °C until transported to the University of Seoul.

### Sediment sampling and PCB analysis

At the NBH and SC sites where fish were collected, surficial sediment samples were collected and measured for total PCBs, as described by Nacci et al. (2002). The concentrations of 18 *ortho*-substituted PCB congeners were detected using microwave extraction, followed by gas chromatography analysis. The environmental Standard Reference Material 1944 (New York/New Jersey Waterway Sediment) certified by the National Institute of Standards and Technology (NIST) was used to validate the analytical methods (Schantz et al. 1997).

### Tissue dissection

Upon arrival at the University of Seoul, fish livers and brains were immediately dissected on ice. The sample groups included NBH and SC fish, and each group was further divided into male and female fish. After dissecting the brains and livers of the fish from each of the four groups, each tissue was split in half and transferred to a cryotube vial. The number of extracted NBH male brains was five, and that of extracted tissue samples from all other groups was six each. The tissues were kept at −80 °C until global DNA methylation and metabolomic analyses were conducted.

### Global DNA methylation

After tissue homogenization, the total DNA was extracted using a DNA extraction kit (NucleoSpin, Macherey-Nagel, Duren, Germany), and the quantity and quality of the extracted DNA were measured using a NanoDrop instrument (ASP-2680, ACTGene, NJ, USA). Global DNA methylation assays were performed using the MethylFlash global DNA methylation 5-mC ELISA Easy Kit (Epigentek, New York, USA) according to the manufacturer’s instructions. Briefly, 100 ng of DNA was added to a 5-methylcytocine (5-mc)-coated well and incubated at 37 °C in the dark for 60 min. The antibody mix (anti-5mc and secondary antibody in the provided ELISA buffer) was then added to each well, and the plate was incubated for 1 h at 37 °C. Subsequently, the absorbance at 450 nm was measured after adding a color developer, and the % of 5-mc was calculated from a standard curve. Appropriate negative and positive controls were used to prepare the standard curve.

### Metabolomics

#### Sample preparation

A 20 mg sample was transferred to a nuclear magnetic resonance (NMR) nanotube, and 25 μL of D_2_O containing 2 mM TSP-d4 (3-(trimethylsilyl) propionic-2,2,3,3-d4 acid sodium salt) was added to the NMR nanotube. The samples were loaded onto a 4 mm nano zirconium rotor. The total volume was adjusted to 45 µL with deuterium oxide to provide a filled lock. The samples also contained 2 mM TSP-d4, as a reference. The lid was capped to close the rotor and marked at the rotor to monitor the spinning speed.

#### NMR experiment

^1^H-NMR experiments were carried out on an Agilent 600 spectrometer (Agilent Technologies, CA, USA) operating at 600.17 MHz equipped with a gH(X) nanoprobe. All data were collected at a spinning rate of 2000 Hz, and the spectra were checked between the water peak and sideband, which coincided with the spin rate. The spectra were recorded at 299.1 K with a spectral width of 9600 Hz, an acquisition time of 2.999 s, a relaxation delay of 1.0 s, 128 scans, and total acquisition times of 13 min and 9 s. The 1D proton NMR spectra were acquired with a CPMG (Carr-Purcell-Meiboom-Gill) pulse sequence to suppress the water signal and macromolecules.

#### Data process

All data were Fourier-transformed and calibrated to TSP-d4 as 0.00 ppm using the Chenomx NMR suite 7.1 professional (Chenomx Inc., Edmonton, Canada). All spectra were processed and assigned using the Chenomx NMR suite 7.1 professional and Chenomx 600 MHz library database. All data were converted to the frequency domain and corrected for phase and baseline, and the TSP-d4 singlet peak was then adjusted to 0.00 ppm. Normalization of the total area of the spectrum was applied to each sample dataset to minimize the effects of variable concentrations among the different samples. Metabolic pathway enrichment with differential metabolites was analyzed using MetaboAnalyst 5.0 (https://www.metaboanalyst.ca/ (accessed May 2023)).

### Statistics and visualization

Statistical significance analyses between groups and visualizations were conducted using R version 4.0.4. When both normality (assessed with the Shapiro-Wilk test) and equal variance between data (assessed with the Bartlett test) were assumed, the significance of the differences between the NBH and SC groups was analyzed using an independent t-test. When normality and/or equal variance was not fulfilled, a nonparametric Wilcoxon rank-sum test was performed. Metabolites that showed statistically significant differences (*p*-value < 0.05) between the SC and NBH groups were determined as differential metabolites.

For metabolomic data, multivariate statistical analyses were performed on all samples using the SIMCA-P + 12.0.1 software package (Umetrics, Umeå, Sweden). Orthogonal projection to latent structure-discriminant analysis (OPLS-DA) was applied to discriminate between the NBH fish and SC fish groups. Additionally, hierarchical clustering with the average linkage method was conducted using R version 4.0.4. The source code and data are available on GitHub at https://github.com/JiwanGV/Killifish-NBH-Metabolites.

## Results

### PCB concentrations in sediments

The total PCB concentration detected in the NBH sediments was 4587 ng/g (approximately 4.6 ppm), which was much higher than the concentration detected in the SC sediments (Table 1). As the detection limit of our methods was 1 ng/g (dry weight), the PCB congeners detected in SC sediments were determined to be present at concentrations less than 1 ng/g. The average sediment PCB concentrations for upper, lower, and outer NBH were 75, 5.1, and 0.2 ppm in 2009, respectively (Nelson and Bergen 2012). Referring to other studies, the total concentration of the 18 PCBs, the same as in this study, ranges from 91,202 to 117,194 ng/g (approximately 91 to 117 ppm) in the livers of adult killifish from NBH (Fritsch et al. 2015; Gräns et al. 2015). These levels in biota were higher than the PCB concentration in sediments in this study.

**Table 1:** PCB concentrations detected in sediments collected from Scorton Creek and New Bedford Harbor

PCB congeners		Concentration ng/g dry wt (SEM)	
		Scorton Creek	New Bedford Harbor
PCB 8	2,4’-Dichlorobiphenyl	ND	50.61 (4.85)
PCB 18	2,2’,5-Trichlorobiphenyl	ND	126.83 (37.01)
PCB 28	2,4,4’-Trichlorobiphenyl	ND	546.85 (192.66)
PCB 52	2,2’,5,5’-Tetrachlorobiphenyl	ND	746.21 (202.48)
PCB 44	2,2’,3,5’-Tetrachlorobiphenyl	ND	232.60 (50.96)
PCB 66	2,3’,4,4’-Tetrachlorobiphenyl	ND	375.07 (33.62)
PCB 101	2,2’,4,5,5’-Pentachlorobiphenyl	ND	495.88 (54.05)
PCB 118	2,3’,4,4’,5-Pentachlorobiphenyl	ND	614.34 (63.75)
PCB 153	2,2’,4,4’,5,5’-Hexachlorobiphenyl	ND	482.18 (49.39)
PCB 105	2,3,3’,4,4’-Pentachlorobiphenyl	ND	187.03 (8.77)
PCB 138	2,2’,3,4,4’,5’-Hexachlorobiphenyl	ND	425.26 (46.58)
PCB 187	2,2’,3,4’,5,5’,6-Heptachlorobiphenyl	ND	62.98 (10.61)
PCB 128	2,2’,3,3’,4,4’-Hexachlorobiphenyl	ND	95.21 (9.87)
PCB 180	2,2’,3,4,4’,5,5’-Heptachlorobiphenyl	ND	79.64 (5.21)
PCB 170	2,2’,3,3’,4,4’,5-Heptachlorobiphenyl	ND	49.17 (3.15)
PCB 195	2,2’,3,3’,4,4’,5,6-Octachlorbiphenyl	ND	6.93 (0.75)
PCB 206	2,2’,3,3’,4,4’,5,5’,6-Nonachlorobiphenyl	ND	8.59 (0.43)
PCB 209	Decachlorobiphenyl	ND	1.42 (0.06)
ΣPCBs		ND	4586.81

Detection limit: 1 ng/g dry wt

*ND* Not detected

### Global DNA methylation

**Fig. 1:**
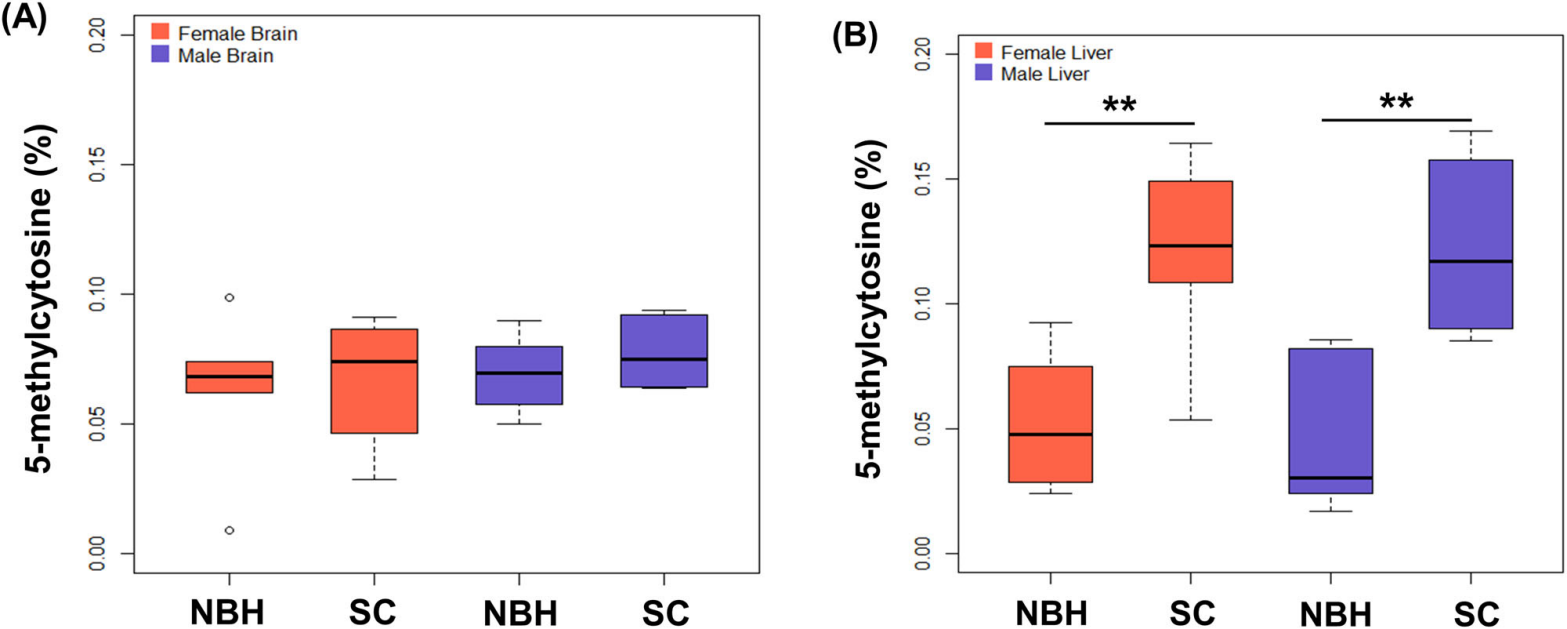
Global DNA methylation levels in brains (**A**) and livers (**B**) of Scorton Creek (SC) and New Bedford Harbor (NBH) fish. Asterisk (*) indicates the significant differences between NBH and SC fish (**p < 0.01)

Global DNA methylation was examined in two tissues and sexes of killifish caught from NHB and SC (Fig. 1). The significant differences in DNA methylation between NBH and SC fish were not observed in brain tissues, but the differences were significant in liver tissues. DNA hypomethylation was observed in the liver of both male and female fish from NBH compared to that from SC.

### Differential metabolites

**Fig. 2:**
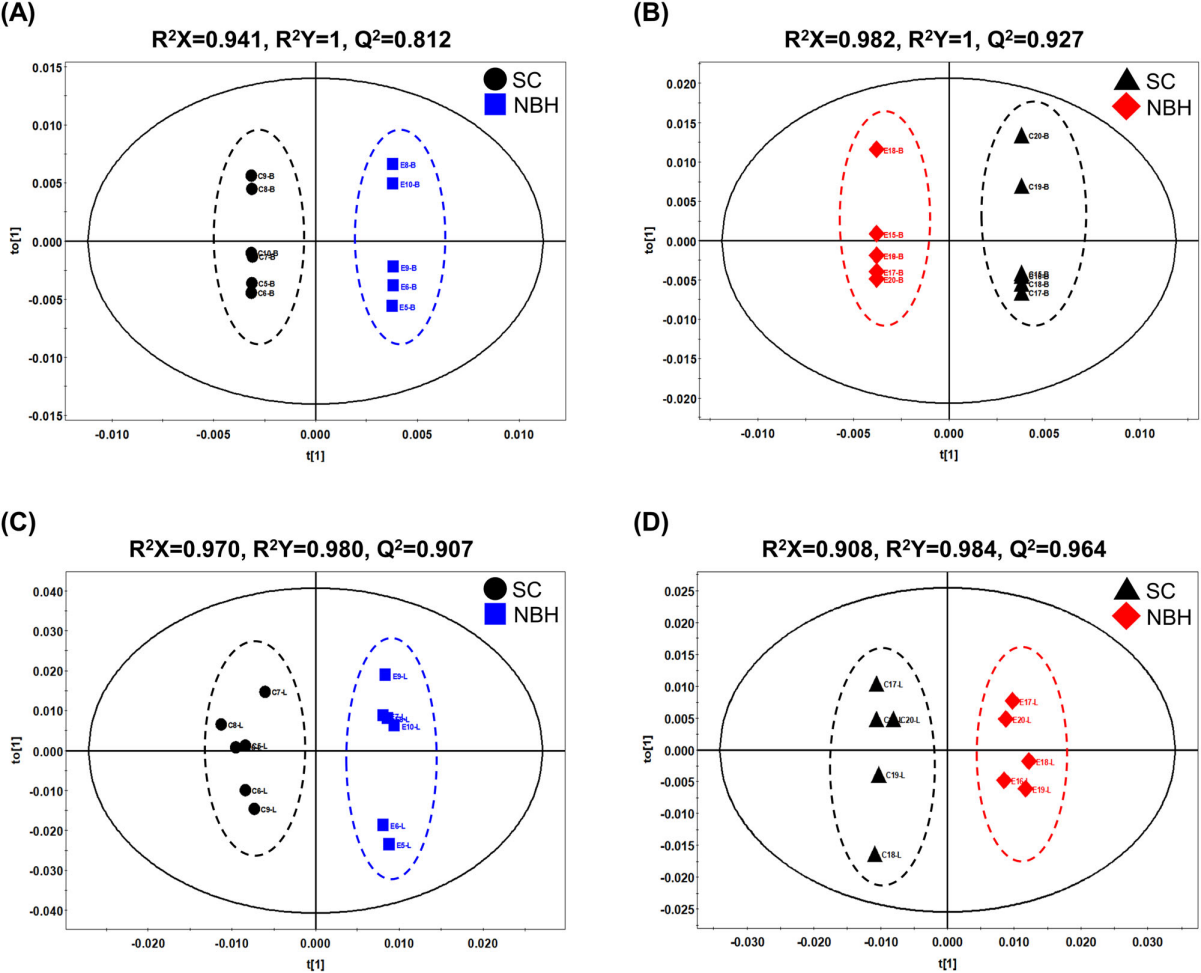
Orthogonal projection to latent structures-discriminant analysis (OPLS-DA) of metabolites detected in brain and liver tissues of Scorton Creek (SC) and New Bedford Harbor (NBH) fish. Male brain (**A**); Female brain (**B**); Male liver (**C**); Female liver (**D**)

NMR-based global metabolomics analysis was conducted on the brain and liver tissues of male and female killifish from NBH and SC. The concentrations of all detected metabolites and fold changes between NBH and SC fish are shown in the supplementary tables (Supplementary Table S1 for metabolites in the brain; Supplementary Table S2 for metabolites in the liver). The OPLS-DA showed the clear class discrimination between the NBH fish and the SC fish in both brain and liver tissues, suggesting that the metabolites of the NBH population were distinct from the SC population (Fig. 2). In addition, the metabolite patterns of NHB fish were substantially different from that of SC fish, as shown in the hierarchical clustering heatmap for the overall metabolite concentration of individual fish (Supplementary Fig. S1). However, there were no different patterns of metabolites between male and female within tissue samples (Supplementary Fig. S2 and Fig. 4A).

**Fig. 3:**
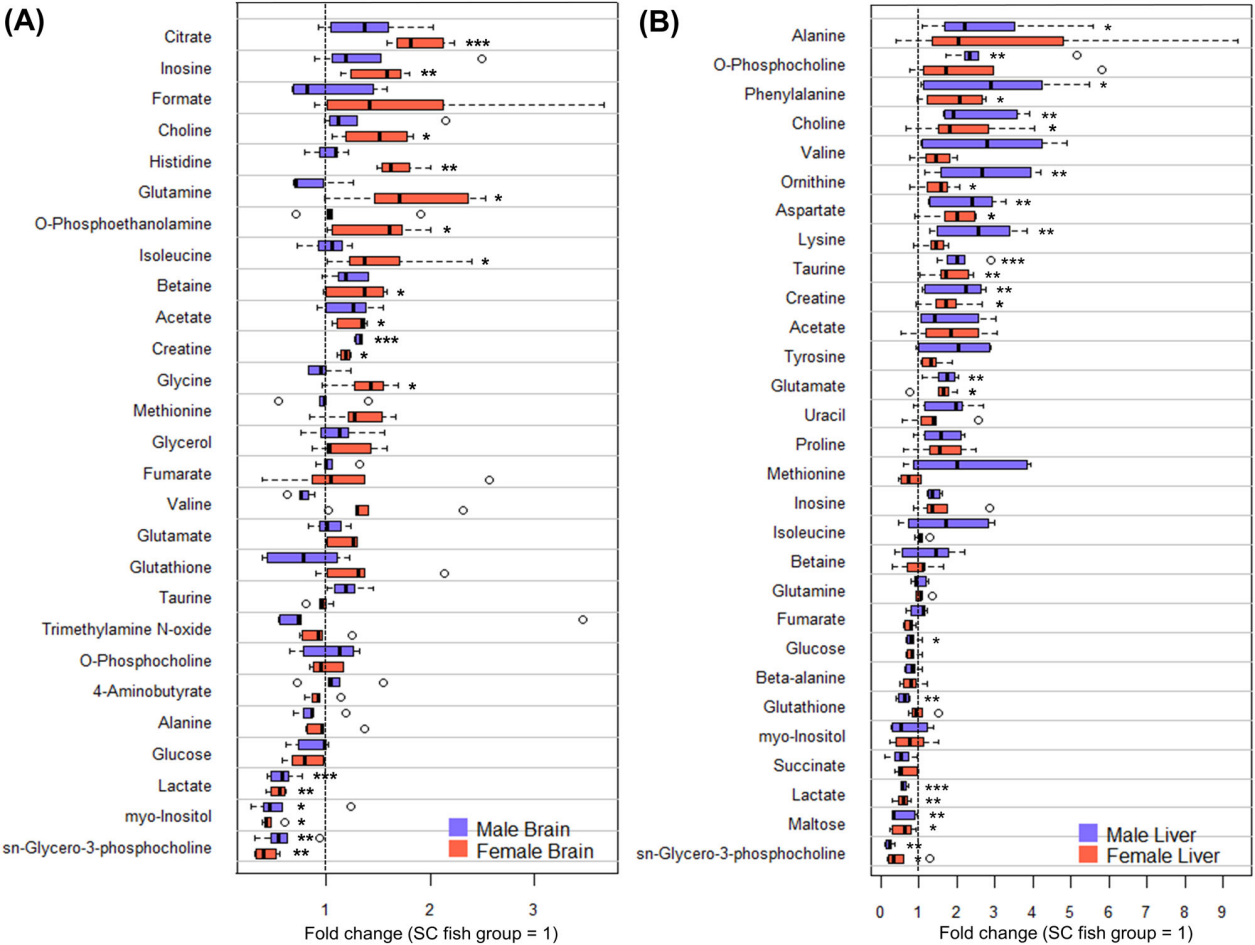
Comparison of fold changes in the concentrations of metabolites detected in (**A**) the brains and (**B**) livers. Asterisk (*) indicates the significant differences between NBH and SC fish (*p < 0.05; **p < 0.01; ***p < 0.001)

**Fig. 4:**
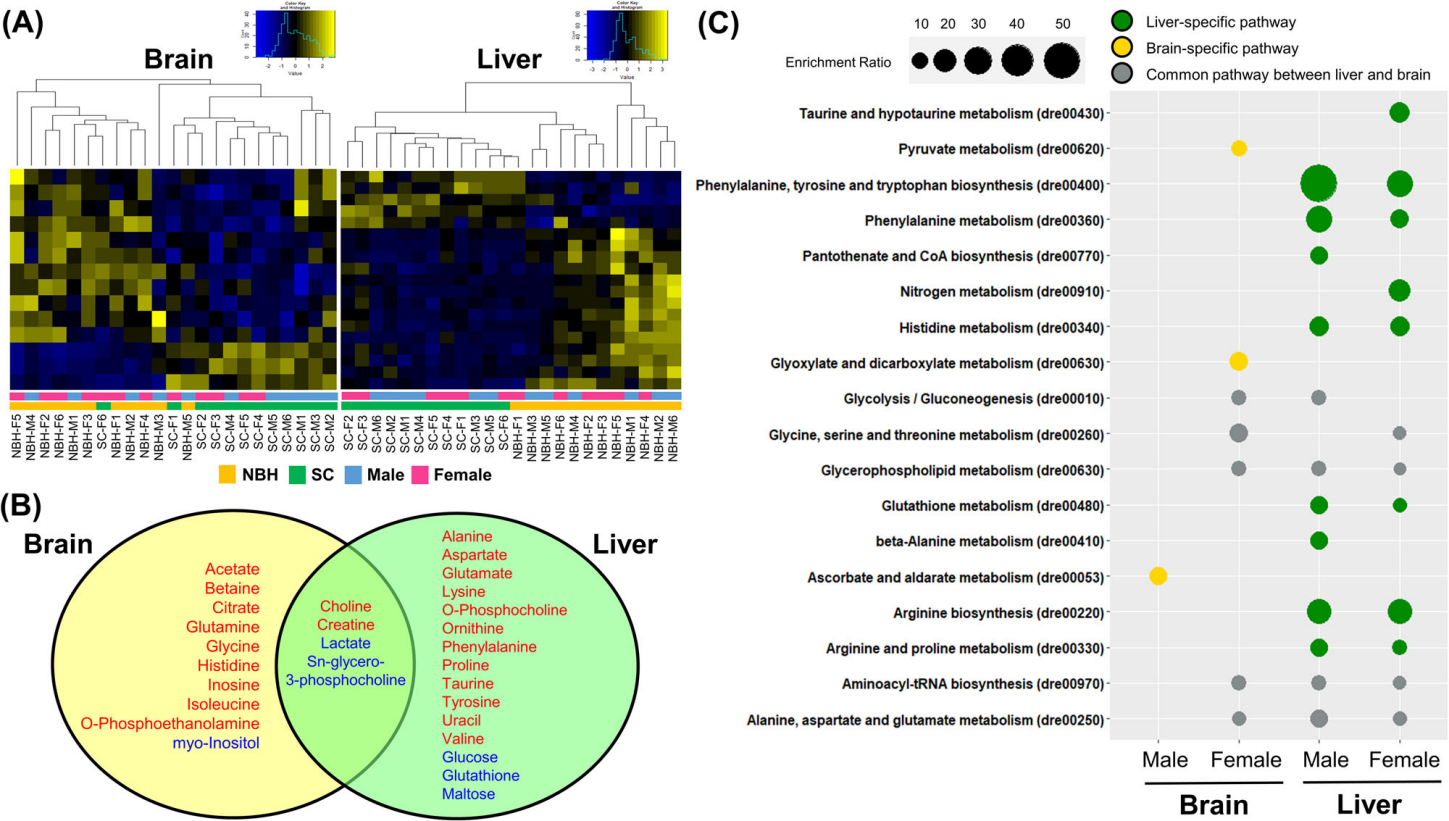
Analysis of differential metabolites between New Bedford Harbor (NBH) and Scorton Creek (SC) fish. **A** Hierarchical clustering heatmap of differential metabolites in brain and liver tissues (SC-M: Male fish from Scorton Creek; SC-F: Female fish from Scorton Creek; NBH-M: Male fish from New Bedford Harbor; NBH-F: Female fish from New Bedford Harbor). **B** Tissue-specific and common metabolites of NBH fish (Red metabolites: the metabolites detected at a higher level than SC fish; Blue metabolites: the metabolites detected at a lower level than SC fish). **C** Pathway enrichment analysis of differential metabolites in brain and liver tissues displaying pathway group (Green circle: liver-specific pathways; Yellow circle: brain-specific pathways; Gray circle: common pathways between two tissues) and enrichment ratio (% = the number of metabolite hits/total number of metabolites in each pathway). The *p*-value of each pathway enrichment is presented in Supplementary Table S3

Among 27 and 29 metabolites detected in our brain and liver samples respectively, differential metabolites from NBH were quantitatively detected in respect to those from SC (Fig. 3 and Table 2). Generally, differential metabolites were 14 in the brain tissue, and 19 in the liver tissue. The detected concentration pattern of these metabolites differed between NBH and SC fish in both brain and liver tissues (Fig. 4A). Four differential metabolites (Choline, creatine, lactate, and sn-glycero-3-phosphocholine) were common in both tissues (Fig. 4B). Among the common metabolites, choline and creatine showed higher levels, and lactate and sn-glycero-3-phosphocholine showed lower levels in NBH fish compared to SC fish. Higher concentrations of choline and o-phosphocholine were detected in the NBH fish group versus the SC fish group (Fig. 3 and Table 2). Moreover, the choline levels in the liver of NBH fish were higher than those in the brain, and the level of o-phosphocholine, a downstream metabolite of choline, was significantly higher in the liver of males with the highest choline levels (Table 2). The concentration of sn-glycero-3-phosphocholine was at the lowest levels in all sample types, and the largest difference was found in the male liver (fold change = 0.24). Except for the common metabolites, 10 were brain-specific and 15 were liver-specific metabolites (Fig. 4B).

**Table 2:** Differential metabolites and their fold changes in NBH fish compared to SC fish

Metabolite	Brain		Liver	
	Female	Male	Female	Male
	**Metabolite with increased fold change**			
Acetate	1.27	NS	NS	NS
Alanine	NS	NS	NS	2.72
Aspartate	ND	ND	1.93	2.27
Betaine	1.31	NS	NS	NS
Choline	1.48	NS	2.13	2.45
Citrate	1.87	NS	ND	ND
Creatine	1.18	1.32	1.75	2.03
Glutamate	NS	NS	1.56	1.69
Glutamine	1.79	NS	NS	NS
Glycine	1.39	NS	ND	ND
Histidine	1.68	NS	ND	ND
Inosine	1.51	NS	NS	NS
Isoleucine	1.51	NS	NS	NS
Lysine	ND	ND	NS	2.53
O-Phosphocholine	NS	NS	NS	2.72
O-Phosphoethanolamine	1.50	NS	ND	ND
Ornithine	ND	ND	1.51	2.71
Phenylalanine	ND	ND	1.97	2.95
Proline	ND	ND	NS	1.59
Taurine	NS	NS	1.81	2.06
Tyrosine	ND	ND	NS	1.97
Uracil	ND	ND	NS	1.81
Valine	NS	NS	NS	2.82
	**Metabolite with decreased fold change**			
Glucose	NS	NS	NS	0.82
Glutathione	NS	NS	NS	0.61
Lactate	0.53	0.58	0.59	0.60
Maltose	ND	ND	0.60	0.53
myo-Inositol	0.45	0.59	NS	NS
sn-Glycero-3-phosphocholine	0.41	0.58	0.49	0.24

*ND* Not Detected, *NS* Not Significant

### Pathway analysis

Pathway enrichment analysis was conducted using the MetaboAnalyst 5.0 platform with the differential metabolites, which showed significant differences in concentration between NBH and SC fish (*p* < 0.05). Several pathways, which included multiple differential metabolites, were identified in the liver, whereas fewer pathways were identified in the brains of male and female fish (Fig. 4C and Supplementary Table S3). The enriched pathways found only in the brain were three carbohydrate metabolisms: Ascorbate and aldarate metabolism, pyruvate metabolism, and glyoxylate and dicarboxylate metabolism. A total of 10 pathways were specifically enriched in the liver. These pathways involved the metabolism of amino acids and cofactors, such as arginine biosynthesis, glutathione metabolism, histidine metabolism, and pantothenate and CoA biosynthesis. The pathways that were commonly found in both the brain and liver included aminoacyl-tRNA biosynthesis, glycine, serine and threonine metabolism, glycerophospholipid metabolism, glycolysis/gluconeogenesis, and alanine, aspartate and glutamate metabolism.

## Discussion

### Liver-specific DNA hypomethylation

We observed significant tissue-specific differences in global DNA methylation. In agreement with the present study, Glazer et al. (2018) reported a significant hypomethylation in the livers of killifish from NBH compared to a reference site. The epigenomic landscape varies across different cell and tissue types, and disease- and trait-associated genetic variants can be linked with tissue-specific epigenomic marks (Roadmap Epigenomics Consortium et al. 2015). The tissue-specific epigenetic differences may be associated with the cell proliferation properties of the tissues. The liver can be more susceptible to the accumulation of DNA mutations, including epimutations, due to its relatively higher proliferation properties than other tissue types (Thompson et al. 2010). Additionally, epigenetic changes can occur in the propinquity of genes, which have the roles in metabolism and metabolic dysregulation (Thompson et al. 2010). The liver is a fundamental organ that performs functions in various physiological processes, including digestion, energetic metabolism, and xenobiotic detoxification. Thus, DNA hypomethylation in the liver may be associated with modified biochemical and metabolic activity. Altered one-carbon energy metabolism in the livers of NBH killifish was proposed as one of the strategies to resist to polluted environments, according to the results of hepatic global 5-methylcytosine levels, DNA methylation machinery gene expression, and differential metabolite concentrations. (Glazer et al. 2018).

The liver is a crucial organ responsible for choline metabolism, and choline is a component of cellular and mitochondrial membranes, as well as the neurotransmitter acetylcholine. Choline can be phosphorylated and subsequently used for the biosynthesis of phospholipid, or it can be oxidized and influence the production of S-adenosylmethionine (SAM) (Corbin and Zeisel 2012). Choline and its metabolite, o-phosphocholine, were enriched in glycerophospholipid metabolism and were detected in higher concentrations in liver tissue (Fig. 4 and Table 2). However, betaine and glycine, which are involved in methionine metabolism, showed no difference between NBH and SC fish, or were not detected (Fig. 3 and Table 2). These results suggest that choline can be used to produce more o-phosphocholine, accompanying a decrease in choline available for betaine and glycine production. In a previous study on NBH killifish, the authors observed higher levels of choline and lower levels of glycine betaine (Glazer et al. 2018). Insufficient glycine and betaine could lead to a reduction in methionine formation from homocysteine and a subsequent decrease in SAM levels (Corbin and Zeisel 2012). As SAM is a major and universal methyl donor, its decrease may cause DNA hypomethylation.

One of the widely reported mechanisms for adaptation to PCBs and PAHs in killifish populations inhabiting Superfund sites is the reduced inducibility of cytochrome P4501A (CYP1A), a downstream gene of the aryl hydrocarbon receptor (AHR) pathway. The analysis of CpG methylation level of CYP1A promoter region did not exhibit any alterations in the liver of killifish caught from the creosote-contaminated Elizabeth River (Timme-Laragy et al. 2005). In a similar line of evidence, Aluru et al. (2011) found no significant differences in the methylation status of CpG sites of AHR1 and AHR2 promoter between the livers of killifish from the PCB-contaminated NBH and those from a reference site.

We propose two hypotheses in terms of epigenetic alterations in NBH killifish. One possibility is that the observed difference in global methylation is a direct result of recent exposure to contaminants, while the adaptation of the NBH population is not associated with epigenetic regulation. Another possibility is that global DNA hypomethylation may be caused by altered levels of metabolites or may participate in regulating the expression of enzyme genes, which could lead to increased levels of differential metabolites (see Fig. 4). To elucidate the role of DNA hypomethylation in transcriptional regulation in the NBH killifish population, it would be beneficial to conduct further analysis of methylated gene regions using more precise technology (e.g., methylation-specific PCR (MSP) or bisulfite sequencing).

### Tissue-specific metabolic pathways

As metabolomics provides a direct “functional readout of the physiological state” of an organism (Gieger et al. 2008), the distinct metabolomic profiles can reflect physiological variations between two populations. In this study, significantly more different metabolites and related pathways were found in the liver tissue of both male and female NBH fish than SC fish, as opposed to the brain tissue (Fig. 4).

#### Urea cycle in the liver

Among the differential metabolites, creatine, ornithine, glutamate, proline, and aspartate are involved in various amino acid metabolism pathways (e.g., arginine and proline biosynthesis, arginine biosynthesis, and nitrogen metabolism), which are closely associated with the urea cycle, an endogenous metabolic removal system in the liver. Similar to this study, Glazer et al. reported a higher level of ornithine and arginine in the NBH killifish population compared to the SC population (Glazer et al. 2018). The urea cycle acts as a detoxification mechanism by converting toxic ammonia into less toxic urea in the liver, which is then excreted (Randall et al. 1989). Most teleost fish are ammonotelic, meaning that they generate ammonia from nitrogen in their liver and excrete it directly through their gills, with smaller amount of urea (Anderson 2001; Randall et al. 1989). However, fish exhibit alterations in urea production and the urea cycle when exposed to or adapted to abnormal environmental stressors. For example, the tilapia fish *Oreochromis alcalicus graham* is able to survive in the alkaline soda lake of Kenya (Lake Magadi) due to its unique ability to excrete nitrogenous waste as urea, unlike other tilapia fish from Sagana river that excrete most of their nitrogenous waste as ammonia (Randall et al. 1989). The Magadi tilapia showed higher levels of ornithine-urea cycle enzymes compared to the Sagana tilapia. This suggests that urea production via ornithine can play a role in the ability to tolerate highly alkaline conditions.

Ammonia in aquatic environments can be originated from a variety of natural sources (e.g., biological nitrogen fixation and nitrogen release from organismal metabolism or decomposition) and anthropogenic sources (e.g., urban sewage discharge and agricultural runoff). One study demonstrated that manipulating pH did not result in any change in toxicity (despite the fact that concentration of the more toxic, unionized ammonia increases at higher pH), thus, ammonia was excluded as a major contributor to the toxicity of NBH sediments (Ho et al. 1997). Moreover, sediments from NBH sites had non-toxic levels of unionized ammonia in their interstitial waters (Ho et al. 2002). Assuming that ammonia in the surrounding environment is not an influencing factor, ammonia can be produced during the metabolism of toxic substances and proteins that fish use for survival and growth. Based on the fact that ammonia is the main metabolic waste product of fish (Francis-Floyd et al. 2022), NBH fish populations may activate the urea cycle to eliminate the ammonia produced in the process of metabolizing xenobiotic compounds and amino acids to survive in stressful conditions (as indicated by the enrichment of many amino acid metabolisms, refer to Fig. 4).

Park and colleagues have identified transcriptional upregulation of genes related to the urea cycle, cytochrome P450, glycogen and glucose metabolism, and nuclear receptors in zebrafish liver spheroids treated with estrogenic chemicals (Park et al. 2022). Furthermore, the concentrations of environmental pollutants, including polychlorinated biphenyls (PCBs), polybrominated diphenyl ethers (PBDEs), polycyclic aromatic hydrocarbons (PAHs), and chlorinated pesticides, were found to be higher in tree swallows from Maumee River relative to those from a reference site, Star Lake (Tseng et al. 2023). Tseng et al. reported that the urea cycle, aspartate metabolism, and arginine and proline metabolism were among the top differentially altered metabolite enrichment pathways in the livers of tree swallow nestlings collected along the Maumee River in Ohio, USA.

#### Glutathione metabolism in the liver

The concentration of glutathione (GSH) was found to be significantly lower only in the liver of male NBH fish (Table 1). Glutathione S-transferase (GST) is a phase II biotransformation enzyme that conjugates glutathione (GSH) to xenobiotic metabolites in the detoxification reaction of xenobiotics. We infer that the lower levels of GSH in NBH fish were due to the utilization of GSH as a substrate for GSTs (Eroglu et al. 2015; Masella et al. 2005; Monteiro et al. 2006). Several studies have observed basal levels of GST activity and increased GST induction in PCB or PAH-resistant killifish, which were caught from contaminated sites. The basal levels of hepatic GST activity were higher in male NBH fish than in male SC fish (Bello et al. 2001). In addition to basal difference, male NBH fish treated with 2,3,7,8-tetrachlorodibenzofuran (TCDF) showed a significant increase in GST activity compared to male SC fish, but female NBH fish did not exhibit the same increase. GST activity can be influenced by hormonal expression, which could account for the sex-specific differences in glutathione levels (Bello et al. 2001).

In another study, the resistant killifish from PAH-contaminated Elizabeth River had elevated hepatic GST levels and activity relative to fish from a less contaminated site and a reference site (Armknecht et al. 1998). Although GSH levels and GST activities in adult killifish vary depending on factors such as sex, tissue, sampling season, and site (mostly the Elizabeth River) (Armknecht et al. 1998; Bacanskas et al. 2004; Bello et al. 2001), the modified glutathione and its metabolism may be involved in the mechanism of tolerance to persistent organic pollutants, such as PCBs and PAHs.

#### Carbohydrate metabolism in the brain

Carbohydrate metabolisms such as glyoxylate and dicarboxylate metabolism, ascorbate and aldarate metabolism, and pyruvate metabolism, were brain-specific enrichment pathways. Citrate, glycine, acetate, and glutamine are involved in glyoxylate and dicarboxylate metabolism, which interconnects with several energy metabolic pathways such as pyruvate metabolism and citrate cycle.

Ascorbate and aldarate metabolism includes *myo*-inositol, which was detected at low concentrations in the brain tissue. *Myo*-inositol is a modulator for multifarious physiological functions as well as an essential nutrient for the growth in aquatic animals. Cui et al. (2022) reviewed the molecular and physiological regulation of *myo*-inositol and stated the beneficial function of this molecule on salinity tolerance, immune system, and stress responses. For example, pretreatment of *myo*-inositol with following copper exposure increased GSH content and GST activities, preventing the fish brain from oxidative damage (Jiang et al. 2014). Based on these previous results, *myo*-inositol may be consumed to provide energy for NBH killifish to survive deleterious conditions or to protect against oxidative stress induced by toxic substances in the environment.

Overall, we identified tissue-specific variations in metabolites and global DNA methylation in killifish populations from the NBH and SC. Although it is widely accepted that the epigenome can act as a mediator between environmental cues and the phenotype of an organism by regulating the transcription of a variety of genes (Cavalieri and Spinelli 2017), studies on epigenetics in killifish populations adapted to environmental pollution have primarily focused on the AHR pathway-related genes. We postulate that liver-specific differential metabolites and DNA hypomethylation or their crosstalk could be factors governing the adaptive responses of wild-caught killifish to chemical contamination. Molecular pathways, particularly the urea cycle and glutathione metabolism found in this study, may also contribute to the resistance of killifish in Superfund sites, and epigenetic mechanisms may involve in this process. However, in addition to pollution factors, the environment in which the two populations inhabited or genetic background can be a major covariate for epigenetic changes and differential metabolic profiles. For example, ecological factors (e.g., biodiversity, food, and geographical traits) can partly influence the epigenome and metabolome. Thus, the analysis of biodiversity and water chemistry characterization in NBH and SC or additional experiments using laboratory-reared animals will help improve our understanding of the mechanisms of adaptation to environmental pollution.

## Conclusion

We identified significant variations in global DNA methylation and metabolite profiles in a contaminant-adapted killifish population from New Bedford Harbor (NBH) compared to killifish from Scorton Creek (SC), a reference site. It is noteworthy that global DNA hypomethylation was evident and liver-specific in the NBH killifish. Differential metabolites differed by tissue and sex, but hierarchical clustering revealed only significant pattern differences between liver and brain tissues, not between males and females. Overall, our results suggest potential metabolic mechanisms associated with the adaptation of killifish surviving in NBH sites and the possible involvement of epigenetic regulation in these mechanisms. However, further analysis of methylated gene regions is required to elucidate the functional role of DNA hypomethylation in the transcriptional regulation and metabolism of NBH populations.

### Supplementary information

Supplementary Figures

Supplementary Tables

## Abbreviation

NBH: New Bedford Harbor
SCH: Scorton Creek
