# Supplementary material for: Differential DNA methylation and metabolite profiling of Atlantic killifish (Fundulus heteroclitus) from the New Bedford Harbor Superfund site
Source: Ecotoxicology. 2024 Jan 6;33(1):22–33. doi: 10.1007/s10646-023-02724-w (PMC10830762; doi:10.1007/s10646-023-02724-w)
Supplement: Supplementary file 1 — Supplementary Figures [file 10646_2023_2724_MOESM1_ESM.pdf]

Supplementary figure

Differential DNA methylation and metabolite profiling of Atlantic killifish (*Fundulus heteroclitus*) from the New Bedford Harbor Superfund site

Jiwan Kim[1], Dawoon Jung[2], Nivedita Chatterjee[1], Bryan Clark[3] Diane Nacci[3], Suhkmann Kim[4] and Jinhee Choi[1*]

[1]School of Environmental Engineering, University of Seoul, 163 Seoulsiripdaero, Dongdaemun-gu, Seoul 02504, Korea

[2]Korea Environment Institute, Division of Environmental Health, Sejong, 30147 Korea

[3]U.S. Environmental Protection Agency, Office of Research and Development, Center for Environmental Measurement and Modeling, Atlantic Coastal Environmental Sciences Division, Narragansett, RI, USA

[4]Department of Chemistry, Center for Proteome Biophysics and Chemistry Institute for Functional Materials, Pusan National University, Busan 46241, Korea

[*]Corresponding author:

Tel: 82-2-6490-2869

Fax: 82-2-6490-2859

E-mail: jinhchoi@uos.ac.kr

Supplementary figure

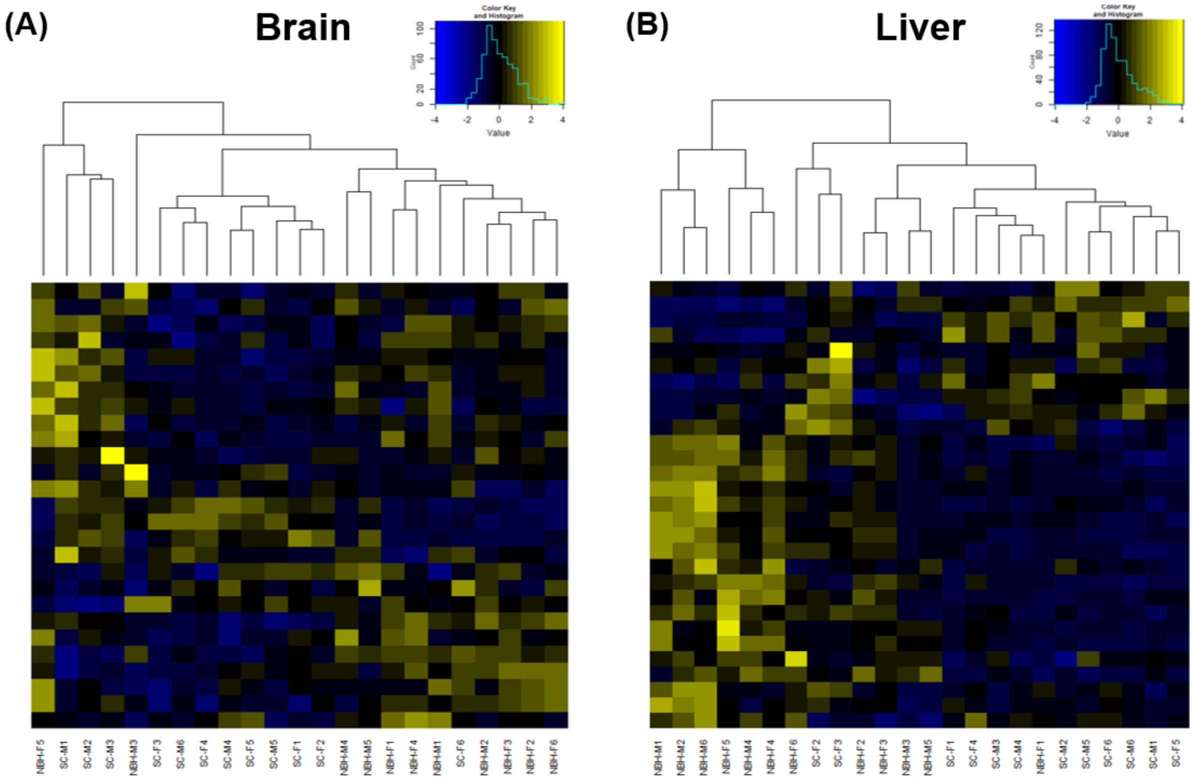

Figure S1. Hierarchical clustering heatmap of all detected metabolites in brain and liver tissues (SC-M: Male fish from Scorton Creek; SC-F: Female fish from Scorton Creek; NBH-M: Male fish from New Bedford Harbor; NBH-F: Female fish from New Bedford Harbor).
